# Preventive child health care at elementary school age: The costs of routine assessments with a triage approach

**DOI:** 10.1371/journal.pone.0176569

**Published:** 2017-04-26

**Authors:** Janine Bezem, Catharina van der Ploeg, Mattijs Numans, Simone Buitendijk, Paul Kocken, Elske van den Akker

**Affiliations:** 1 Municipal Health Service Gelderland-Midden, Arnhem, The Netherlands; 2 Netherlands Organisation for Applied Scientific Research TNO, Leiden, The Netherlands; 3 Public Health and Primary Care Department, Leiden University Medical Centre, Leiden, The Netherlands; 4 Education Office, Imperial College, London, United Kingdom; 5 Medical Decision-Making Department, Leiden University Medical Centre, Leiden, The Netherlands; King's College London, UNITED KINGDOM

## Abstract

**Background:**

Triage in Preventive Child Health Care (PCH) assessments could further the efficient use of human resources and budgets and therefore make extra care possible for children with specific needs. We assessed the costs of routine PCH assessments with and without triage for children aged 5/6 years and 10/11 years. In a triage approach, PCH assistants conduct pre-assessments to identify children requiring follow-up assessments by a physician or nurse. In the usual approach, all children are assessed by a physician and an assistant (children aged 5/6 years) or a nurse (children aged 10/11 years).

**Methods:**

All the direct costs of conducting routine PCH assessments with the triage and usual approach were assessed using a bottom-up micro-costing approach. In four PCH services in the Netherlands, two using triage and two the usual approach, professionals completed questionnaires about time spent on assessments, including time related to non-attendance at assessments, the referral of children and administration.

**Results:**

The projected costs for PCH professionals working on PCH assessments amounted to €5.2 million per cohort of 100,000 children aged 5/6 years in the triage approach, and €7.6 million in the usual approach. The projected costs in both approaches for children aged 10/11 years were about €4 million per 100,000 children.

**Conclusion:**

The triage approach to PCH resulted in a projected cost reduction of about one-third, compared with usual practice, for routine assessments by physicians of children aged 5/6 years. There are minimal cost savings in the group of children aged 10/11 years when nurses are involved and so other considerations such as workforce shortages would be required to justify a change to a triage approach. Further research is needed to investigate the differences in costs of care after the completion of the routine assessments.

## Background

There is a growing realisation that health services for children, including preventive health services, should be managed efficiently. The delivery of preventive child health assessments needs to be more efficient because of organisational challenges in terms of limited financial resources, staff shortages and the high workloads of physicians and nurses, and the need to use workforce competences better [1]. Moreover, changing the organisation of preventive child health care (PCH) will create opportunities to spend more time on current health issues such as mental health problems, preventing violence, lifestyle-related problems and inequities in child health [1–4]. Changing the workforce skill mix by using triage and shifting tasks of health professionals, in particular in primary and emergency health care services, could be a way of delivering cost-conscious health services without negatively affecting the quality of health care [5–8]. However, there is a lack of research looking at the costs of changing the skill mix in primary care, including PCH, and of role changes involving workers other than physicians and nurses [9–11]. Research shows that shifting tasks from physicians to nurses in primary care has a positive impact on patient satisfaction [9,12]. Research into the efficiency of shifting tasks from physicians to nurses shows that nurses spend more time on assessments and return consultations [13,14]. However, given their lower salaries, this could still result in a reduction in the costs of provided care. The current study used assessment duration and the hourly wages for the different disciplines to investigate costs.

Many countries provide preventive child health care services for vaccinations and a pre-defined schedule of assessments for the early detection of health problems in children [4]. These services are often performed by nurses. In the Dutch PCH programme, all children may undergo seventeen routine assessments between birth and 18 years of age: thirteen in the first four years of life (in well-child clinics), and four between the ages of 4 and 18 years (in school health services). These assessments are conducted by PCH physicians and nurses until the age of 6 years and by PCH nurses for the older age groups. When PCH physicians and nurses identify problems, they decide whether to refer children for additional assessments by the PCH, or to external services [15]. Rising health care costs have challenged the Dutch PCH to find innovative organisational models for the efficient delivery of preventive health services. In the approach as usual, all children aged 5 to 6 years are assessed by a PCH physician with support from an assistant. There are two possibilities for children aged 10 to 11 years: in one PCH service the children are assessed by a nurse and in the other the nurse receives support from a PCH assistant. These assessments are conducted, in the case of younger children, in the presence of parents. As the benefits of triage and task-shifting are known in other sectors in health care, we investigated whether these can also be found in preventive health care [5–8]. A new two-step procedure was developed for children aged 4 to 18 years involving triage and the shifting of tasks from PCH physicians and nurses to PCH medical assistants [15]. Children are first assessed by a PCH assistant who receives training at the vocational education level that focuses specifically on medical issues. The pre-assessment of the children is conducted using a strict protocol and includes the completion of questionnaires by parents and school teachers, and a face-to-face screening (that includes, for example, screening of growth, hearing and vision). Only children who have missed the pre-assessment and children with suspected health care needs are referred for follow-up assessment by a PCH physician or nurse. The nature and complexity of the health problems determine which professional is needed for follow-up assessment: follow-up assessments for medical and developmental disorders are performed by physicians, while follow-up assessments for psychosocial problems and lifestyle issues are mostly performed by nurses. The pre-assessment by the PCH assistants takes place at schools in the absence of parents but with parental consent. Follow-up assessments by a physician or nurse are conducted in the presence of the children’s parents. The triage approach leads to less involvement of physicians and nurses in the routine assessments, creating time for them to provide additional consultations tailored to children’s specific health needs.

Other studies examining the triage approach to child health assessments showed that the attendance levels with this approach matched those for the usual approach and that it seemed to detect health problems as effectively as the usual approach [15–17]. Significantly fewer children were referred for additional assessment by PCH or for treatment to external health services in the triage approach [15]. Further research is needed to compare the efficiency of care delivered by the triage approach with usual practice [16]. The aim of this study was to compare the costs of conducting preventive child health assessments with a triage approach and the usual approach. We limited this study to the costs for the system of routine PCH assessments.

## Methods

PCH services in the Netherlands offer routine health assessments for children in elementary schools from two age groups, namely 5/6 and 10/11 years. A bottom-up micro-costing approach was used to analyse the costs of the triage and usual approaches at these ages. The measure used was the salary costs for the PCH professionals who conducted the preventive assessments. We distinguished between these salaries and the costs associated with the time taken by parents to attend the assessments of their children, two elements which together make up the costs for society [18]. Other costs such as the costs of consumables, rent and utilities were not included as these are expected to be comparable for both approaches.

### Data collection

PCH physicians, nurses and assistants recorded the time they spent on conducting the assessments (face-to-face (FtF) and non-FtF, in terms of contact by email or telephone), on referring children and on administration. If children failed to attend the appointments, the professionals estimated the time taken to complete the non-attendance protocol and the lost time that could not be spent on other productive professional activities. In the case of care as usual, each physician and each nurse recorded the time needed to conduct FtF assessments using a stopwatch for ten of their children per age group (5/6 and 10/11 years), and each assistant timed 20 children per age group. In the triage approach, each assistant recorded the time duration of 20 FtF assessments using a stopwatch. A total of 518 triage assessments and 529 assessments with usual care were timed by PCH professionals. In the triage approach, physicians and nurses conduct follow-up assessments of specific problems such as behaviour, hearing or weight problems. Because most problems targeted by follow-up assessments are too rare to be measured repeatedly, respondents were asked not to use a stopwatch but to estimate the average time needed for both FtF and non-FtF assessments of these specific problems on the basis of their experience with multiple previous cases [18]. PCH professionals completed questionnaires about time spent on assessments that covered time recorded using a stopwatch and estimated time. Time records were obtained from 32 physicians (18 triage, 14 usual approach), 31 nurses (16 triage, 15 usual approach) and 22 assistants (13 triage, 9 usual approach). In addition, for usual care as well as triage, all PCH assistants asked four parents in each age group how much time the parents needed to travel to and from the assessment, including waiting time.

The measurements with a stopwatch were first averaged for each individual professional, and these mean outcomes per professional were then averaged for all the professionals in the same discipline. The estimates of average time needed to assess specific problems were averaged for the professionals in the same discipline. The time taken by parents was calculated by averaging the responses from the parents.

Data collection took place in the last quarter of 2012.

To study the costs of routine health assessments we used data from a larger study of the effects of the triage approach which was performed in two PCH services using the triage approach and two PCH services with similar demographic characteristics which used the usual approach [16]. Each PCH service covers a population of around 125,000 children annually aged between 0 and 18 years. We selected a sample of 1008 children (from 20 schools) assessed using the triage approach and a sample of 986 children (from 21 schools) assessed with the usual approach. The samples were randomly selected from elementary schools which were stratified for socio-economic status (low, middle and high status) and the urban or rural area selected.

### Cost per assessment

The average time spent on the assessments and the salaries for each discipline was used to calculate the costs of the assessment [19]. Costs were indexed from 2011 to 2013 using the Dutch Consumer Price Index [20]. The costs per hour were €104 for physicians, €62 for nurses and €49 for assistants. The time spent by parents on attending the assessment, travelling and waiting were valued at an hourly rate of €13.60 on the basis of the cost of replacing household care (price level 2013) [18].

### Analyses

**Fig 1:**
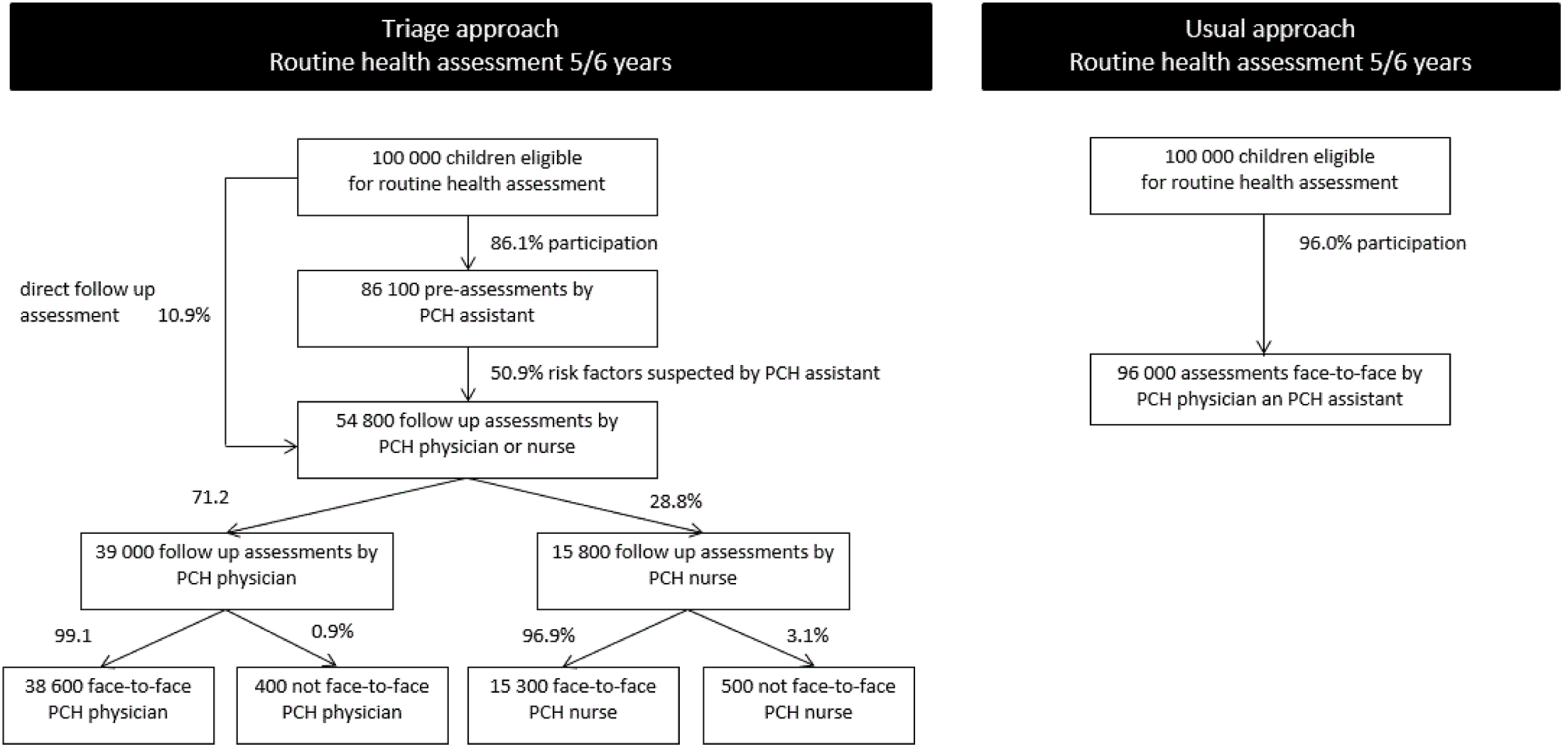
Flow chart outlining the assessments of a theoretical cohort of 100,000 children aged 5–6 years, triage and usual approach.

**Fig 2:**
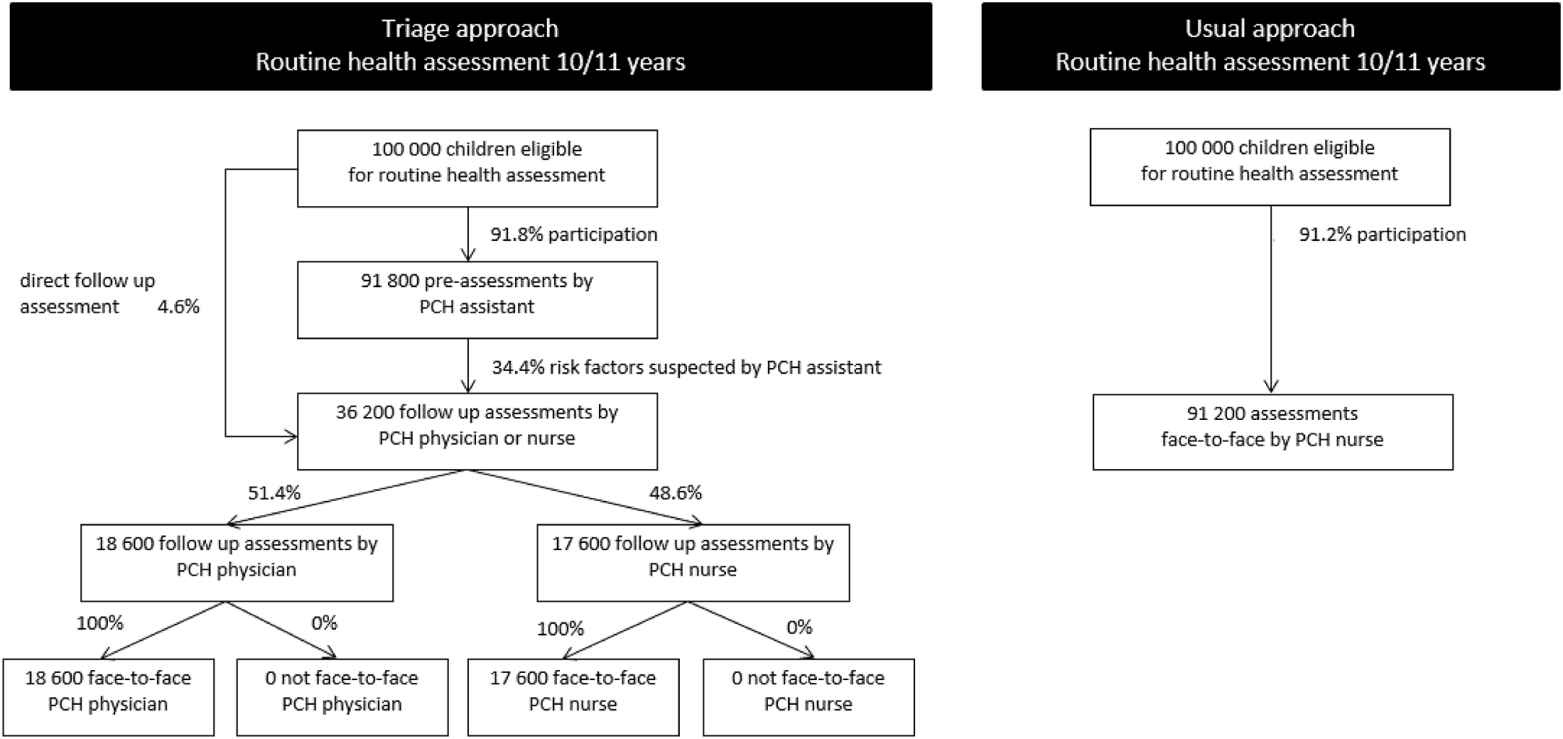
Flow chart outlining the assessments of a theoretical cohort of 100,000 children assessed aged 10–11 years, triage and usual approach.

To compare the costs of the triage and usual approach, we calculated the projected costs for a theoretical cohort of 100,000 children. Attendance rates for the pre-assessments and follow-up assessments and the referral rate to follow-up assessments in the triage approach—and the attendance and referral rates for assessments in the usual approach—(see Figs 1 and 2) were measured as part of the larger study of the effects of the triage approach using a study cohort of 1897 children [16]. We used these rates to determine the numbers of the different types of assessments needed for a theoretical cohort of 100,000 children. By multiplying the numbers of the different types of assessments by the costs, we obtained the total projected costs for both approaches. The total projected costs of parental attendance were calculated on the basis of the number of assessments in combination with the average time needed by parents to accompany their child.

Sensitivity analyses were performed to assess whether the cost calculations were sensitive to uncertainty in our assumptions and estimates. We used the socio-economic status of the schools (extracted from national census statistics established on the basis of the postal code for the school address) to determine the effect that socio-economic status had on the results. In the primary analysis, we assumed that there was no difference in socio-economic status. To determine whether this assumption was justified, cost calculations were also made for both socio-economic status groups (low and medium/high). Another sensitivity analysis was conducted of the time estimates for follow-up assessments in the triage approach. A few PCH professionals estimated that a large amount of time would be required on average for some specific problems. In this sensitivity analysis, we examined the impact on total costs when we used maximum amounts of time for follow-up assessments: 60 minutes for FtF, 30 minutes for non-FtF and 30 minutes for administration. When professionals thought more time was needed, those estimates were disregarded (‘missing’ result).

### Ethics statement

The Medical Ethics Committee of Leiden University Medical Centre approved this study.

## Results

Tables 1 and 2 show the projected costs associated with the time spent by the various professionals on preventive health assessments in the triage and usual approaches for children aged 5/6 and 10/11 years on the basis of a theoretical cohort of 100,000 children a year.

**Table 1:** Costs (2013 €) associated with the time spent by PCH professionals on preventive assessments of child health in the triage approach and the usual approach for children aged 5/6 years on the basis of a theoretical cohort of 100,000 children per year.

	Triage approach (N = 100,000)				Usual approach (N = 100,000)		
	Pre-assessment by PCH assistant	Follow-up assessment by PCH physician	Follow-up assessment by PCH nurse	Total	Assessment by PCH assistant	Assessment by PCH physician	Total
Number of children attending assessment							
Face-to-face (N)	86 100^1^	38 600^2^	15 300^2^		96 000^3^	96 000^3^	
Not face-to-face (N)	-	400^2^	500^2^		-	-	
Mean time spent by professionals on assessment per child (min)	25.6	specified in Table 3	specified in Table 3		25.4	28.5 / 33.1^4^	
Total time spent by professionals on assessments (hrs)	36 600	20 900	9 500	67 100	40 700	46 900	87 600
Costs of conducting assessments (euro)	1 800 000	2 173 000	588 000	4 561 000	1 998 000	4 869 000	6 867 000
Number of non-attended assessments (N)^5^	23 300	31 600	12 800		24 400	24 400	
Mean time related to non-attendance per child (min)^6^	5.8	14.1	19.5		7.8	13.0	
Total time related to non-attendance (hrs)	2 300	3 700	2 100	8 000	3 200	5 300	8 500
Costs non-attendance (euro)	111 000	384 000	128 000	623 000	155 000	549 000	705 000
Total costs of conducting assessments including non-attendance (euro)				5 184 000			7 572 000

^1^On the basis of an attendance rate of 86.1%. Some of the 100,000 children receive a follow-up assessment directly without a pre-assessment.

^2^On the basis of the number of children attending pre-assessment (86,100), the percentage of children referred to a follow-up assessment (50.9%) and the percentage of children receiving a follow-up assessment without pre-assessment (10.9%). In the follow-up assessment, 71.2% of the children were seen by the PCH physician and 28.8% by the PCH nurse.

^3^On the basis of an attendance rate of 96.0%.

^4^28.5 minutes if no referral to additional assessment by PCH or external health services was needed; 33.1 minutes if a referral to additional assessment was necessary.

^5^More than one non-attendance per child is possible.

^6^Time to conduct non-attendance protocol and lost time for professionals.

**Table 2:** Costs (2013 €) associated with the time spent by PCH professionals on preventive assessments of child health in the triage approach and the usual approach for children aged 10/11 years on the basis of a cohort of 100,000 children per year.

	Triage approach (N = 100,000)				Usual approach^1^			
					Assessment by PCH assistant and nurse (N = 100,000)			Assessment by PCH nurse (N = 100,000)
	Pre-assessment by PCH assistant	Follow-up assessment by PCH physician	Follow-up assessment by PCH nurse	Total	Assessment by PCH assistant	Assessment by PCH nurse	Total	Assessment by PCH nurse
Number of children attending assessment								
Face-to-face (N)	91 800^2^	18 600^3^	17 600^3^		91 200^4^	91 200^4^		91 200^4^
Not face-to-face (N)	-	0^3^	0^3^		-	-	-	-
Mean time spent by professionals on assessment per child (min)	22.1	specified in Table 3	specified in Table 3		10.3	27.7 / 40.6^5^		36.2 / 43.5^6^
Total time spent by professionals on assessments (hrs)	33 900	11 300	10 400	55 500	15 600	46 400	62 000	57 500
Costs of conducting assessments (euro)	1 664 000	1 171 000	642 000	3 477 000	767 000	2 869 000	3 636 000	3 555 000
Number of non-attended assessments (N)^7^	15 400	10 200	9 600		36 100	36 100		36 100
Mean time related to non-attendance per child (hrs)^8^	5.8	14.1	19.5		7.8	14.3		14.8
Total time related to non-attendance (hrs)	1 500	2 400	3 100	7 000	4 700	8 600	13 300	8 900
Costs non-attendance (euro)	73 000	248 000	194 000	515 000	230 000	533 000	763 000	550 000
Total costs of conducting assessments including non-attendance (euro)				3 992 000			4 399 000	4 105 000

^1^Two different methods can be distinguished for the usual approach: a combined assessment by a PCH nurse and an assistant, and an assessment by a PCH nurse only.

^2^On the basis of an attendance rate of 91.8%. Some of the 100,000 children received a follow-up assessment directly without a pre-assessment.

^3^On the basis of the number of children attending pre-assessment (= 91 800), the percentage of children referred to a follow-up assessment (34.4%) and the percentage of children receiving a follow-up assessment without pre-assessment (4.6%). In the follow-up assessment 51.4% of the children were assessed by the PCH physician and 48.6% by the PCH nurse. All children were assessed face-to-face.

^4^On the basis of an attendance rate of 91.2%.

^5^27.7 minutes if no referral to additional assessment by PCH or external health services was needed; 40.6 minutes if a referral to additional assessment was necessary.

^6^36.2 minutes if no referral to additional assessment by PCH or external health services was needed; 43.5 minutes if a referral to additional assessment was necessary.

^7^More than one non-attendance per child is possible.

^8^Time to conduct non- attendance protocol and lost time for professionals.

In a theoretical cohort of 100,000 children, the time spent by professionals on assessments of children aged 5/6 years was 67,100 hours in the triage approach and 87,600 in the usual approach (Table 1). In a theoretical cohort of 100,000 children aged 10/11 years, the total time spent on assessments was 55,500 hours in the triage approach, 62,000 hours in the usual approach if assessments were performed by both the assistant and nurse and 57,500 hours if the assessment was conducted by a nurse alone (see Table 2). A valuation was made of the time spent by the different professionals on the basis of their hourly rates. That valuation resulted in total projected costs (excluding non-attendance) of €4,561,000 in the triage approach and €6,867,000 in the usual approach for children aged 5/6 years. The projected costs were lower for children aged 10/11 years: €3,477,000 in the triage approach, €3,636,000 in the usual approach if assessments are performed by both a nurse and an assistant and €3,555,000 if the assessment is performed by a nurse alone.

The time and costs associated with non-attendance (the time needed to implement the non-attendance protocol and lost time for professionals) are shown in Tables 1 and 2. The projected costs per 100,000 children associated with non-attendance in the triage approach vary from €515,000 to €623,000 depending on the age group. In the usual approach, the costs vary from €550,000 to €763,000.

The time needed to conduct follow-up assessments in the triage approach and the extra time needed to refer children to additional assessments by PCH or to external services was estimated by physicians and nurses (see Table 3). The time spent on follow-up assessments targeting specific problems of children in the triage approach, as estimated by the physician, was between 19.4 minutes for vision problems to 85.3 minutes for child abuse in face-to-face contacts. In the case of face-to-face follow-up assessments performed by a nurse, the estimated time ranged from 10.5 minutes for musculoskeletal reasons to 73.4 minutes for child abuse (see Table 3).

**Table 3:** Average estimated time spent by professionals on follow-up assessments in the triage approach (minutes).

	Triage approach					
	Follow-up assessment by PCH physician			Follow-up assessment by PCH nurse		
	Face-to-face^1^	Not face-to-face^2^	Extra time for referring^3^	Face-to-face^1^	Not face-to-face^2^	Extra time for referring^3^
Musculoskeletal	20.1	9.0	6.8	10.5	3.7	7.5
Cognitive development	37.2	18.9	22.0	22.8	17.5	13.8
Weight	22.9	12.7	7.2	24.0	19.4	12.9
School problems	40.8	22.1	21.7	36.3	28.7	25.8
Child abuse	85.3	32.9	32.2	73.4	36.1	41.7
Lifestyle	27.9	17.3	11.2	26.1	20.8	15.0
Length	19.6	11.1	8.6	18.0	10.0	15.0
Motor development	28.5	11.8	8.1	14.3	8.3	-
Eyesight	19.4	10.1	6.4	12.7	7.3	5.3
Hearing	20.5	9.8	7.4	10.7	7.3	4.0
Psychosocial development	47.1	24.4	30.8	36.5	29.1	29.5
Speech and language development	25.2	11.3	8.9	13.4	9.3	4.7
Truancy	58.5	23.9	24.6	26.3	20.0	15.0
Cleanliness	28.6	15.7	9.2	28.9	22.9	11.3
Not otherwise specified	26.3	6.4	9.7	23.5	14.6	6.5

^1^Time needed for ‘Face-to-face follow-up assessment’ includes time for preparation, consultation and reporting results.

^2^Time needed for ‘Non-face-to-face follow-up assessment’, in terms of contact by email or telephone, includes time for preparation, unanswered calls, discussions and reporting results.

^3^Time needed for referral to additional assessments by PCH, or to external services.

**Fig 3:**
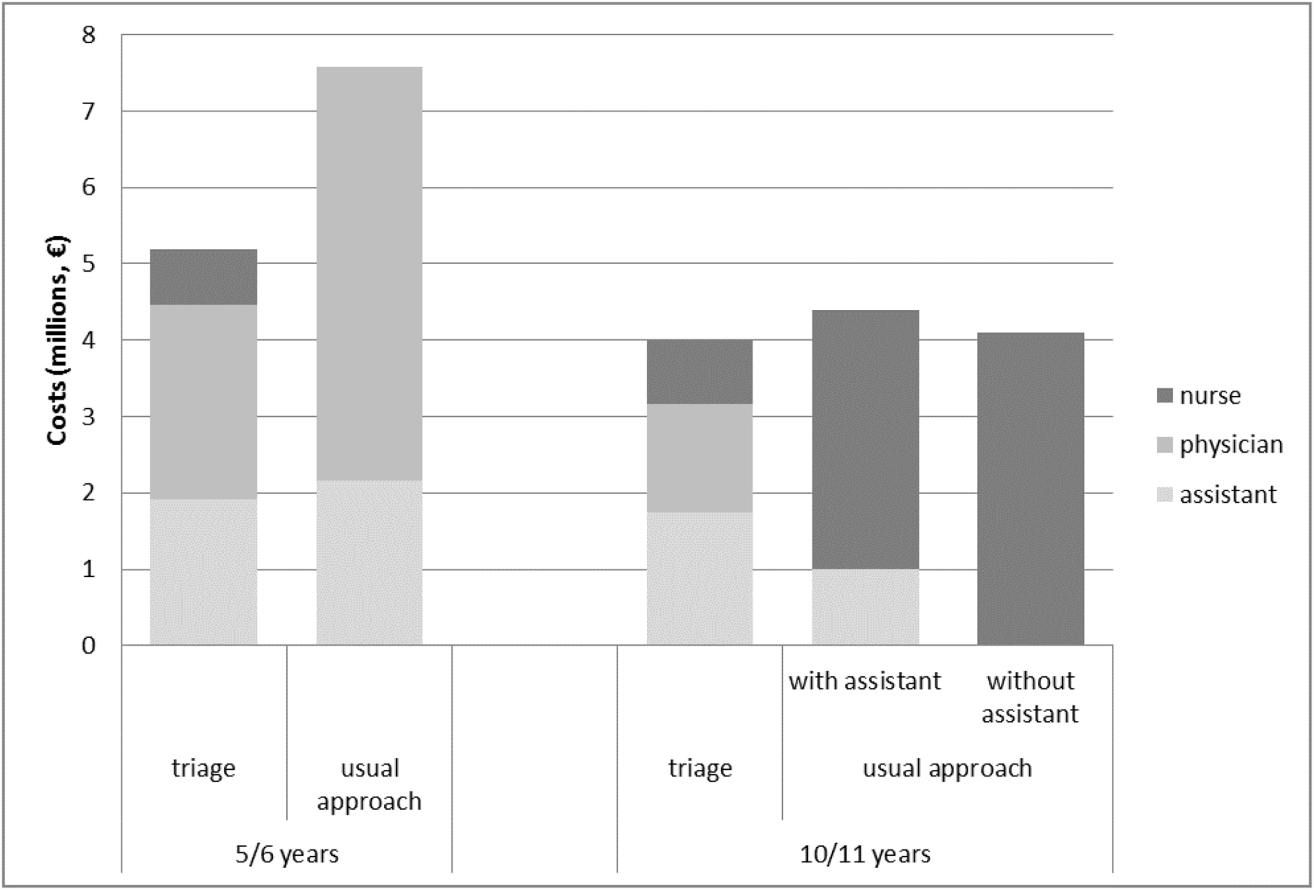
Projected costs (2013 €) of the deployment of professionals for routine health assessments of children aged 5/6 and 10/11 years, triage and usual approaches.

The total projected costs of assessments by PHC professionals (including non-attendance) are €5,184,000 per theoretical cohort of 100,000 children aged 5/6 years in the triage approach, and €7,572,000 in the usual approach. In children aged 10/11 years, the triage approach costs €3,992,000, and the usual approach costs €4,399,000 if the assessments are performed by both a nurse and an assistant and €4,105,000 if the assessment is performed by a nurse alone. The different professionals involved, and the costs of the deployment for the routine assessments of the two approaches, including the costs of non-attendance, are shown in Fig 3. The difference is presented for the two age groups.

The projected costs of parental attendance at assessments, including waiting and travelling time, are shown in Table 4. The projected costs of the triage approach, in which parents only accompany their child if there is a follow-up assessment, are €428,000 for children aged 5/6 years; these costs amount to €1,434,000 in the usual approach. In the case of parents of children aged 10/11 years, the costs are €297,000 in the triage approach and €1,027,000 and €971,000 in the usual approach depending on whether or not the nurse receives support from a PCH assistant.

**Table 4:** Projected costs (2013 €) associated with the time required for parental attendance at the assessments of their children aged 5/6 and 10/11 years on the basis of a theoretical cohort of 100,000 children per year.

	Triage approach	Usual approach	
	Pre-assessment by PCH assistant and assessment by PCH physician or nurse (N = 100,000)	Assessment by PCH assistant and physician/nurse^2^ (N = 100,000)	Assessment by PCH nurse (N = 100,000)
Total time associated with parental attendance^1^ (hrs)			
Children aged 5/6 years	31 500	105 400	-
Children aged 10/11 years	21 800	75 500	71 400
Costs of parental attendance (euro)			
Children aged 5/6 years	428 000	1 434 000	-
Children aged 10/11 years	297 000	1 027 000	971 000

^1^On the basis of the number of children attending assessment (see Tables 1 and 2), the time per assessment (see Tables 1 and 2 and Additional file 1), a mean travelling time of 8.1 minutes (one way) and a mean waiting time of 2.9 minutes.

^2^5/6 years: PCH assistant and physician; 10/11 years: PCH assistant and nurse.

The sensitivity analysis in which we made a distinction between children with a low socio-economic status and children with a medium or high socio-economic status, and the sensitivity analysis in which we placed an upper limit on the time needed for a follow-up assessment in the triage approach led to only marginal differences (€0.30-€2.03 per child) in the results presented and did not affect the conclusions with regard to the comparison of the triage and usual approaches.

## Discussion

This paper describes a comparison of the costs of routine assessments in preventive child health care (PCH) in a triage approach and an approach-as-usual. We found a cost reduction of about one-third for assessments of children aged 5/6 years when the triage approach was used rather than the usual approach. A minimal cost reduction was found in the group of children aged 10/11 years. This difference in the cost reduction for the two age groups can be explained by the time spent by physicians and nurses on assessments for the two age groups (see Fig 3).

In the triage approach for children aged 5/6 years, the cost reduction is attributable to the lower level of physician involvement in the assessment of children in combination with the same level of deployment of PCH assistants with relatively low salary costs by comparison with the usual approach. The cost reduction for children aged 10/11 years can be attributed to the costs required for pre-assessments by PCH assistants in the triage approach (which are lower than the costs of assessments by a nurse in the usual approach). However, this reduction in costs was almost offset by the higher costs of the follow-up assessments by physicians or nurses in the triage approach. Furthermore, we found a reduction in the projected costs of parental attendance at assessments with the triage approach that is attributable to the absence of parents at pre-assessments in the triage approach. This finding applies to both age groups.

### Strengths and weaknesses of the current study

A strength of our study is that we asked a large sample of PCH professionals working with both approaches to measure the main components of the assessments precisely with a stopwatch because this improves the internal validity of the results. This was not done in the follow-up assessments looking at potential health or psychosocial problems as part of the triage procedure because the majority of the problems were too rare to be measured repeatedly. To assess the duration of these problem-specific assessments, the respondents were asked to estimate the mean time spent on the specific assessments (see Table 3). The direction of the possible bias caused by these estimates is not clear. Another strength is that we conducted sensitivity analyses to assess the possible effect of the socio-economic status of the children and the maximum time spent on the follow-up assessments and that we found that the marginal differences did not affect the conclusions with regard to the comparison of the triage and usual approaches. Another strength is that the four PCH services in this study used the same protocolled screening and registration methods, reducing the possibility of reporting bias. A limitation is that the outcomes of the triage approach may have been affected by a difference in the duration of the use of the triage and usual approaches. The triage approach was introduced a few years ago, whereas the PCH services in the approach-as-usual group had been working with this approach for a long time. It can reasonably be expected that triage will have a larger impact on cost efficiency when the triage approach has been in place for a longer period of time. We limited this study to the use of human resources and efficiency, but we have not measured the costs of safeguarding the quality of care in terms of the training and supervision of professionals.

### Implications for practice and directions for future research

Health system issues—in terms of increasing pressures on limited resources and shortages of physicians and nurses—require the development of new organisational models for health care delivery. The outcomes of this study suggest that a triage approach may be a promising way of improving efficiency in countries with systems of preventive child care services delivered by physicians, who are mostly active for the younger age group in well-baby clinics. On the other hand, our study showed that minimal costs savings are obtained with a triage approach for preventive child care services delivered by nurses. Other arguments may also be relevant for the use of the triage approach, such as improvements in the use of the competences of professionals and workforce shortages. Since PCH assessments are typically straightforward and consist to a large extent of routine protocolled screening activities conducted by dedicated PCH professionals, it is reasonable to argue that the organisation of PCH can be changed by optimising the workforce skill mix. The impact on health outcomes resulting from task-shifting depends on the complexity of tasks, degree of autonomy, and level of education and competences of the professionals involved [21, 22]. Investments in training and supervision for the various professionals are therefore necessary to safeguard the quality of care when introducing a triage approach [12]. PCH assistants are typically less expensive to train and to employ than physicians and nurses. However, in the triage approach, PCH assistants need more training and supervision because they take on some of the tasks of the physicians and they work independently. As well as cutting costs, the shifting of tasks from PCH physicians and nurses to assistants is intended to give physicians and nurses more time to provide additional PCH assessments tailored to children’s specific health needs and to respond to requests from parents, school professionals or young people themselves. Another benefit of the triage approach is the reduction in time needed by parents to accompany their children to assessments as they do not attend the pre-assessments in the triage approach. On the other hand, the absence of parents during the assessments may result in less involvement with the PCH professionals.

This study was confined to an analysis of the costs of conducting PCH routine assessments. We did not report on the costs arising after the identification of health concerns by PCH during assessments, an example being the costs of health care involving medical specialists. Further research is needed to investigate the differences between the additional costs of referrals to additional assessment by PCH or to external services in the two approaches. It will be useful to learn more about which children are missed in the two approaches and the long-term health outcomes to further determine the cost-effectiveness of the implementation of the triage approach for preventive assessments of child health. Finally, we did not examine the quality of care, including parent satisfaction, in the two approaches.

## Conclusions

The triage approach to PCH resulted in a projected cost reduction of about one-third, compared with usual practice, for routine assessments by physicians of children aged 5/6 years. There are minimal cost savings in the group of children aged 10/11 years when nurses are involved and so other considerations such as workforce shortages would be required to justify a change to a triage approach. Further research is needed to investigate the differences in costs of care after the completion of the routine assessments.
