# Correction: Preventive child health care at elementary school age: The costs of routine assessments with a triage approach

**DOI:** 10.1371/journal.pone.0183068

**Published:** 2017-08-07

**Authors:** Janine Bezem, Catharina van der Ploeg, Mattijs Numans, Simone Buitendijk, Paul Kocken, Elske van den Akker-van Marle

The sixth author’s name is incorrect. The correct name is Elske van den Akker-van Marle. The correct citation is: Bezem J, van der Ploeg C, Numans M, Buitendijk S, Kocken P, van den Akker-van Marle E (2017) Preventive child health care at elementary school age: The costs of routine assessments with a triage approach. PLoS ONE 12(4): e0176569. https://doi.org/10.1371/journal.pone.0176569
